# Effects of improved ultrafiltration on serum level of IL-6 and TNF-a, HCT, and cardiopulmonary function in patients with extracorporeal circulation in valve replacement

**DOI:** 10.5937/jomb0-54272

**Published:** 2025-07-04

**Authors:** Geng Ning, Lili Fu, Guangwei Zhou

**Affiliations:** 1 Tangshan Workers Hospital, Department of Anesthesiology, Tangshan, Hebei, China

**Keywords:** cardiopulmonary bypass, extracorporeal circulation, inflammatory response, ultrafiltration, valve replacement, kardiopulmonalni bajpas, ekstrakorporalna cirkulacija, inflamatorni odgovor, ultrafiltracija, zamena zalistaka

## Abstract

**Background:**

To investigate the effects of modified ultrafiltration in extracorporeal circulation valve replacement surgery.

**Methods:**

A total of 62 patients with valvular disease who underwent valve replacement were included. They were randomly divided into the conventional ultrafiltration group (CUF group, n=31) and the modified ultrafiltration group (MUF group, n=31). The hematocrit (Hct) values, volume of pleural fluid drainage at 24 hours after operation, Intensive Care Unit (ICU) stay time, postoperative 24-hour blood loss, bank blood usage, postoperative 24-hour urine volume, ventilator support time, cardiac function indexes, postoperative changes of respiratory function, and levels of inflammatory factors in both groups were compared.

**Results:**

After ultrafiltration, the MUF group showed higher Hct value and reduced volume of pleural fluid drainage, blood loss, bank blood usage, urine volume and ventilator support time 24 hours after operation compared with the CUF group (P<0.05). After surgery, left ventricular ejection fraction (LVEF) levels were elevated, and those in the MUF group were higher than those in the CUF group. Left ventricular end-diastolic diameter (LVEDD) and heart rate (HR) were decreased in both groups after surgery. They were lower in the MUF group than in the CUF group (P<0.05). After ultrafiltration, the OI value in the MUF group was higher, and the alveolar-arterial oxygen partial pressure gradient (P (A-a)O2) value was lower than the CUF group (P<0.05). The plasma concentrations of interleukin 6 (IL-6) and tumour necrosis factor-alpha (TNF-a) were increased after cardiopulmonary bypass (CPB) in both groups and then decreased after ultrafiltration, and IL-6 and TNF-a levels in MUF group were lower than those in CUF group (P<0.05).

**Conclusions:**

MUF attenuates the postoperative systemic inflammatory response, reduces the lung injury caused by CPB, and improves the lung function of patients in the early postoperative period, which benefits patient recovery after surgery and is valuable in heart valve replacement.

## Abstract (sr)

**Uvod:**

Cilj je bio da se istraže efekti modifikovane ultra filtracije tokom zamene srčanih zalistaka u uslovima ekstrakorporalne cirkulacije.

**Metode:**

U istraživanje je uključeno 62 pacijenta sa oboljenjem zalistaka koji su podvrgnuti zameni zalistaka. Pacijenti su nasumično podeljeni u grupu sa konvencionalnom ultrafiltracijom (CUF grupa, n=31) i grupu sa modifikovanom ultrafiltracijom (MUF grupa, n=31). Upoređivane su vrednosti hematokrita (Hct), količina pleuralnog izliva 24 sata nakon operacije, trajanje boravka u jedinici intenzivne nege (ICU), postoperativni gubitak krvi tokom 24 sata, upotreba date krvi putem transfuzije, količina urina 24 sata nakon operacije, vreme ventilacione podrške, pokazatelji srčane funkcije, postoperativne promene respiratorne funkcije i nivoi inflamatornih faktora u obe grupe.

**Rezultati:**

Nakon ultrafiltracije, MUF grupa je pokazala višu vrednost Hct i smanjenu količinu pleuralnog izliva, gubitka krvi, upotrebe transfuzije, količine urina i trajanja ventilacione podrške u poređenju sa CUF grupom (P<0,05). Nakon operacije, nivo izbacivanja leve komore (LVEF) se povećao, a vrednosti u MUF grupi bile su vi{e nego u CUF grupi. Prečnik leve komore na kraju dijastole (LVEDD) i srčana frekvencija (HR) smanjili su se u obe grupe nakon operacije, pri čemu su vrednosti bile niže u MUF grupi nego u CUF grupi (P<0,05). Nakon ultrafiltracije, vrednost oksigenacijskog indeksa (OI) je bila vi{a, dok je gradijent parcijalnog pritiska kiseonika između alveola i arterija (P (A-a)O2) bio niži u MUF grupi nego u CUF grupi (P<0,05). Koncentracije interleukina 6 (IL-6) i faktora tumorske nekroze alfa (TNF-a) u plazmi povećane su nakon kardio -pulmonalnog bajpasa (CPB) u obe grupe, a zatim su smanjene nakon ultrafiltracije, pri čemu su nivoi IL-6 i TNF-a u MUF grupi bili niži nego u CUF grupi (P<0,05).

**Zaključak:**

MUF smanjuje postoperativni sistemski inflamatorni odgovor, umanjuje oštećenje pluća izazvano CPB-om i poboljšava funkciju pluća kod pacijenata u ranom postoperativnom periodu. Ova metoda doprinosi oporavku pacijenta nakon operacije i ima veliki značaj u zameni srčanih zalistaka.

## Introduction

Valvular heart disease is a common chronic cardiovascular disease caused by structural or functional changes in the heart valve and failure in normal blood circulation [1]. Valvular heart disease has increased in recent years [2]. Surgery has been demonstrated to be effective and is recommended for treating this disease. Valve replacement is mainly applied by replacing the failing heart valve with an artificial valve [1] [3]. Extracorporeal circulation provides extracorporeal life support and time to manage acute cardiac or respiratory failure, and cardiopulmonary bypass (CPB) is a well-known type of extracorporeal circulation [4] [5]. However, the application of CPB is limited by pulmonary dysfunction in patients with severe valvular heart disease before operation [6]. CPB can also trigger an inflammatory response, which may lead to acute lung injury, aggravate pulmonary dysfunction, and seriously affect the prognosis of patients [7] [8] [9]. Moreover, CPB can cause hemodilution that contributes to tissue perfusion and dysfunction of end organs [10]. Thus, developing effective strategies to reduce the adverse effects caused by CPB during surgery is imperative.

Ultrafiltration used in CPB has been revealed to filter out excess water in the body, elevate the hematocrit (Hct) and colloid osmotic pressure, reduce levels of inflammatory factors in circulation, prevent pulmonary oedema and improve postoperative pulmonary function [11]. Compared with conventional ultrafiltration, modified ultrafiltration has a more reasonable connection mode and adequate ultrafiltration with improved hemodynamic and organ function in the pediatric population. In contrast, its function in adult patients still requires further investigation [12]. Previous studies have probed into some aspects of modified ultrafiltration on adult patients after CPB [13]. Systematic research is needed to compare the effects of modified ultrafiltration and conventional ultrafiltration on adult patients with valve replacement under CPB. Therefore, this study explored the effects of modified ultrafiltration in extracorporeal circulation valve replacement surgery.

## Materials and methods

### Patients

Sixty-two patients with valvular disease who underwent valve replacement from March 2022 toFebruary 2023 were included. Patients and their families signed informed consent for this study. The same team of surgeons, anesthesiologists, and perfusionists performed all procedures.

### Inclusion and exclusion criteria

Inclusion criteria: (1) Diagnosed of pure aortic valve disease or pure mitral valve disease or pure aortic valve disease combined with mitral valve disease; (2) Aged between 18–75 years; (3) Informed consent was obtained from the patients and their families; (4) American Society of Anaesthesiologists (ASA) physical status was classified as II or III, left ventricular ejection fraction (EF) 40%; (5) New York Heart Association (NYHA) classification of cardiac function II or III.

Exclusion criteria: (1) Severe liver and kidney dysfunction; (2) Unable to cooperate; (3) Myocardial infarction within 3 months; (4) Coronary artery disease and chronic obstructive pulmonary disease; (4) Hematogenic and immune system disease; (5) Patients with severe heart failure and malignant arrhythmia; (6) Pregnancy.

### Sample size

The GPower V3.1 software [14] was used to calculate the sample size under the power value of 0.8, alpha value of 0.05 and the effect size of about 80%, allocation ratio of 1:1 [15]. A total of 52 patients are needed, and each group was allocated 31 patients to improve the power of this study.

### Randomization

The participants were randomly divided into the conventional ultrafiltration group (CUF group, n=31) and the modified ultrafiltration group (MUF group, n=31) in a 1:1 ratio using the random number table method. The Statistical Product and Service Solutions (SPSS) software generated a random number table, and the starting point was set as the first number. The 62 patients were numbered. Those with odd numbers were allocated to the MUF group, and those with even numbers were allocated to the CUF group. The allocation probability was equal among all participants.

### Intervention

### Anesthesia and CPB methods

After local anaesthesia, the left radial artery was punctured, and the direct blood pressure was monitored. The endotracheal intubation was conducted and connected with a ventilator for mechanical ventilation. The anaesthesia was maintained by intravenous and inhalation anaesthesia. Compound electrolyte injection and hydroxyethyl starch 130/0.4 sodium chloride injection were used as prefilling solutions for extracorporeal circulation, and the crystal gel ratio was 1:1. Mild hypothermia (32–35°C) was maintained during CPB. Intermittent 4:1 cold cardioplegic solution containing blood perfusion was used for myocardial protection every 30 minutes. CPB was performed with a Stockert-C artificial heart-lung machine (STOCKERT, Germany), SX18 membrane oxygenator (Terumo, Japan) and domestic tubing.

### Respiratory management method

The ventilation was performed with positive gap pressure, tidal volume of 6–8 mL/kg, inspired oxygen concentration of 30%–60%, positive end-expiratory pressure of 5 cm H_2_O, and respiratory rate of 35 to 45 mmHg of end-tidal carbon dioxide.

### Ultrafiltration method

Patients in the CUF group received conventional ultrafiltration. The outlet end of the arterial microplug filter was connected to the inlet end of the ultrafiltration filter, and the outlet end was connected to the reflux chamber. The ultrafiltration time was determined according to the plane and Hct of the reflux chamber of the blood reservoir, and the machine blood was discharged at the end of ultrafiltration, followed by the routine intravenous infusion for retransfusion.

Patients in the MUF group received modified ultrafiltration. The input end of the ultrafilter was connected with the »T« shape of the arterial circuit, and the output end was connected with the blood reservoir. The ultrafilter pump controlled the blood flow. After the intracardiac procedure, ultrafiltration was started to remove excess water from the circulation. After CPB was stopped, the vena cava cannula was removed immediately, and the machine blood was back into the venous circuit. The output end of the ultrafilter was connected to the internal jugular vein puncture needle (16G). The ultrafiltration flow rate was kept at 10–15 mL/kg·min^-1^ until the blood reservoir ran dry. The main pump controlled the residual machine blood in the oxygenator to replenish the body volume and maintain hemodynamic stability. With the addition of an appropriate amount of Ringer’s lactate solution in the blood reservoir, the machine blood was injected into the body, followed by ultrafiltration time for 15–20 min. The aortic cannula was removed when the internal circulation was low-volume. A small amount of residual machine blood in the oxygenator and extracorporeal circulation pipeline was further concentrated through the ultrafilter and the internal jugular vein under the drive of the ultrafilter pump and then immediately transfused back into the body.

### Primary and secondary outcomes

(1) Bank blood usage, postoperative 24-hour urine volume and ventilator support time in the CUF and MUF groups were compared.

(2) Cardiac function indexes, including left ventricular ejection fraction (LVEF), left ventricular enddiastolic diameter (LVEDD) and heart rate (HR), were measured in both groups.

(3) Tidal volume (TV), respiratory rate (F), ventilator airway peak pressure (Ppeak), respiratory pause pressure (Ppause), inspired oxygen concentration (FiO_2_) and arterial blood gas analysis at each time point were recorded before CPB, at the end of CPB, and after ultrafiltration. Oxygenation index (OI) and alveolar-arterial oxygen partial pressure gradient (P (A-a)O_2_) were calculated based on the above indicators and blood gas results.

(4) The Hct values of both groups were measured before CPB, at the end of CPB, and after ultrafiltration.

(5) The volume of pleural fluid drainage 24 hours after operation, ICU stay time and postoperative 24-hour blood loss in the CUF and MUF groups were compared.

(6) Arterial blood was collected before CPB, at the end of CPB, and after ultrafiltration for blood gasanalysis. The levels of interleukin-6 (IL-6) and tumour necrosis factor-α (TNF-α) in the plasma were measured by ELISA method using commercial assay kits (Human IL-6 ELISA Kit, #PI330; Human TNF-α ELISA Kit, #PT518) obtained from Beyotime (Shanghai, China) following manufacturer’s protocol.

### Statistical analysis

Data analysis was performed using SPSS 10.0 statistical software, and data were shown as the mean ± standard deviation. The difference between the CUF and MUF groups was analyzed using an independent t-test. χ^2^ test was used to compare the qualitative variables between groups. P<0.05 was statistically significant.

### Ethical consideration

This study was approved by the Ethics Committee of our hospital and registered at the Chinese Clinical Trial Registry (Registration number: 3445768).

## Results

### Clinical characteristics of all participants

A total of 62 patients who underwent valve replacement from March 2022 to February 2023 in our hospital were included in this study. The mean age of the participants was 65.24±6.51. In the CUF group, 58% were male (n=18) and 42% were female (n=13). In the MUF group, 55% were male (n=17) and 45% were female (n=14). Among all participants eligible in this study, 50% underwent mitral valve replacement, 33.9% underwent aortic valve replacement, and 16.1% received double valve replacement. There was no significant difference in gender, weight, course of disease, age and type of operation between the two groups (P>0.05, Table 1).

**Table 1:** General data of patients in both groups.

Characteristics	CUF group<br>(n=31)	MUF group<br>(n=31)	P
Gender<br>(male/female)	18/13	17/14	>0.05
Average age<br>(years)	65.28±6.53	65.20±6.48	>0.05
Average course of<br>disease (years)	4.92±1.53	4.98±1.56	>0.05
Weight (kg)	58.26±6.05	58.31±6.08	>0.05
Type of operation			>0.05
Mitral valve<br>replacement	15	16	
Aortic valve<br>replacement	10	11	
Double valve<br>replacement	6	4	

### Primary outcome

### Bank blood usage, postoperative 24-hour urine volume and ventilator support time in both groups

**Figure 1:**
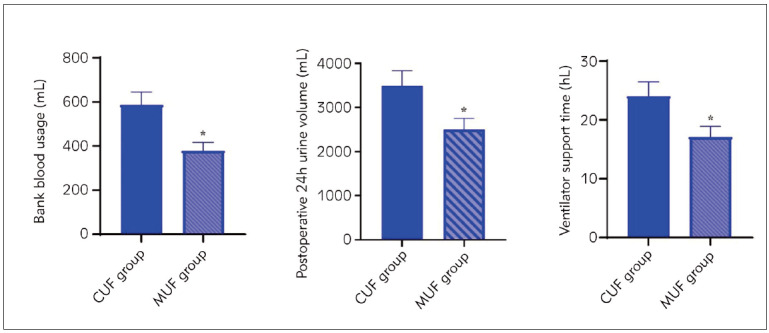
Receiver Operating Curves for prediction of negative SUVmax (SUVmax<1) of ACE and chitotriosidase.

As shown in Figure 1, the bank blood usage in the MUF group was 379.64±38.02 ml and was significantly lower than that in the CUF group (586.73±58.65 mL). The postoperative 24-hour urine volume was significantly lower in the MUF group (2500.64±250.37) compared with the CUF group (3487.12±345.23). Moreover, we also found that the ventilator support time of the MUF group (17.15±1.74 h) was lower than that of the CUF group (24.06±2.43 h).

### Cardiac function indexes in both groups

**Figure 2:**
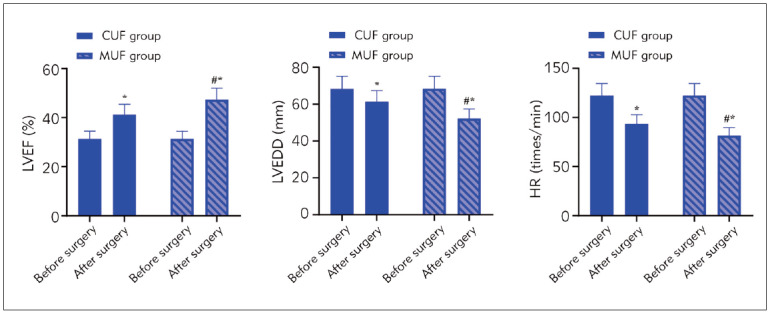
Cardiac function indexes in both groups. *P<0.05, compared with before surgery, #P<0.05, compared with CUF group.

Before surgery, LVEF, LVEDD, and HR levels showed no significant difference between the CUF and MUF groups (P>0.05). After surgery, LVEF levels were elevated and higher in the MUF group than in the CUF group. Meanwhile, LVEDD and HR levels were reduced in both groups after surgery, and those in the MUF group were lower compared to the CUF group (P<0.05, Figure 2).

### Pulmonary function indexes in both groups

Postoperative changes in respiratory function in both groups were monitored. The results showed that after ultrafiltration, the OI value in the MUF group was significantly reversed and was higher relative to the CUF group (P<0.05), while the P (A-a)O_2_ value in the MUF group was lower relative to the CUF group (P<0.05, Table 2).

**Table 2:** Patient respiratory function in both groups.

Group	Time	OI<br>(mmHg)	P (A-a)O2<br>(mmHg)
CUF<br>group	Before CPB	450.23±45.26	17.23±1.75
	After CPB	335.09±34.01	256.28±25.64
	After<br>ultrafiltration	362.38±36.27	368.95±36.78
MUF<br>group	Before CPB	450.29±45.19	17.25±1.73
	After CPB	335.18±33.87	256.21±25.62
	After<br>ultrafiltration	433.74±43.35*	238.47±23.85*

### Adverse event

**Figure 3:**
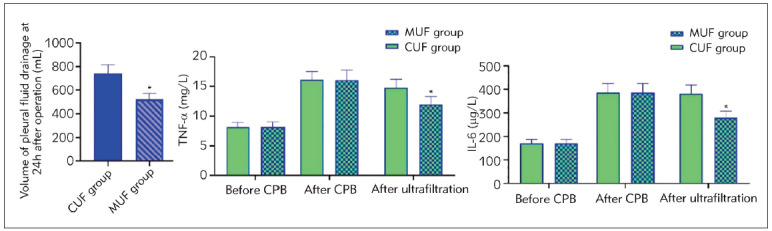
Volume of pleural fluid drainage at 24 hours after operation and inflammation response in both groups. *P<0.05.

Compared with the CUF group, the volume of pleural fluid drainage at 24 hours after operation was 740.28±74.23 mL in the CUF group, which was significantly higher than the 522.21±52.26 ml in the MUF group (Figure 3). Indition, the levels of inflammatory factors in both groups were compared. The plasma concentrations of IL-6 and TNF-α were increased after CPB in both groups. They showed a significant reduction in the MUF group after ultrafiltration, while those in the CUF group were not significantly changed (P<0.05, Figure 3).

### Secondary outcome

### Hct, blood loss, and ICU needs in the two groups

**Figure 4:**
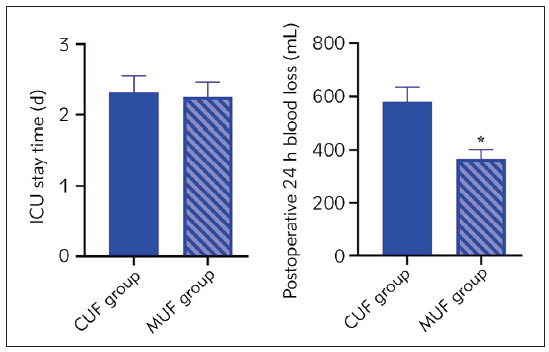
ICU stay time and postoperative 24-hour blood loss in both groups. *P<0.05.

Before and after CPB, we found no significant difference in Hct values between CUF and MUF groups (P>0.05). After ultrafiltration, the Hct value in the MUF group was significantly higher than the CUF group (P<0.05, Table 3). Moreover, the MUF group showed less ICU stay time and postoperative 24-hour blood loss than the CUF group (P<0.05, Figure 4).

**Table 3:** Patient respiratory function in both groups.

Group	Time	Hct (%)
CUF group	Before CPB	34.01±3.42
	After CPB	20.92±2.13
	After ultrafiltration	32.03±3.35
MUF group	Before CPB	34.08±3.43
	After CPB	21.09±2.15
	After ultrafiltration	38.97±3.87*

## Discussion

Patients with heart valve disease usually have a long course of disease and different degrees of cardiac and respiratory dysfunction before surgery [16]. Heart valve replacement surgery under CPB is an effective method for treating severe heart valve disease [17]. However, excessive blood dilution, ischemia-reperfusion injury, accumulation of various metabolites, low temperature and release of various inflammatory mediators commonly occur during cardiac surgery with CPB and can cause pulmonary dysfunction, increase pulmonary vascular resistance and decrease alveolar gas exchange function, prolonging the recovery time of cardiopulmonary function of patients, and increasing the incidence of postoperative complications with life-threatening risks such as adult respiratory distress syndrome (ARDS) [18] [19].

Modified ultrafiltration is reported to effectively concentrate blood and alleviate the adverse effects caused by CPB [20]. Additionally, similar to the principle of glomerular filtration, modified ultrafiltration filters out the water and soluble small molecules from the blood by convection under the action of transmembrane, improving the Hct level in the blood to a certain extent and reducing the postoperative blood loss and the amount of reservoir blood, without increasing the CPB time and being restricted by CPB [21]. Niu et al. [22] have also demonstrated that optimized ultrafiltration significantly elevates the Hct level in children after CPB. In this study, the outcomes demonstrated that after ultrafiltration, the Hct value decreased after CPB was restored by the ultrafiltration in both MUF and CUF groups and that in the MUF group was significantly higher relative to the CUF group (Table IIII), which was consistent with the previous findings. The improvement of Hct and the removal of excess water in the body during ultrafiltration can reduce tissue oedema and improve the symptoms of hypoxia in the body [23], suggesting that the modified ultrafiltration could better improve the oxygenation degree and tissue oedema in the body. Lee et al. [24] have also demonstrated that ultrafiltration improves blood coagulation, attenuates the effects of CPB-induced hemodilution, and increases post-CPB bleeding. However, studies have also suggested that the extension of modified ultrafiltration duration does not alter the ICU stay and time of discharge [25] [26]. In our study, the volume of pleural fluid drainage at 24 hours after operation, postoperative 24-hour blood loss, bank blood usage, postoperative 24-hour urine volume and ventilator support time were lower in the MUF group compared to the CUF group (Figure 1). The ICU stay showed no significant difference between the CUF and MUF groups, while the postoperative 24-hour blood loss was lower in the MUF group than in the CUF group (Figure 4). The difference with previous findings may be caused by the significantly younger age of the participants enrolled in the previous studies and the different control groups used. Moreover, the small sample size and single-centre design may limit the generalization of our results, and the incidence of postoperative events was not monitored for an extended period. Future studies must compare the effects of two interventions in a large sample size and over extended periods. Despite these limitations, our study’s results indicated that modified ultrafiltration could effectively reduce the extravasation of body fluids, filter out excess water in the body after CPB shutdown, and significantly reduce bank blood usage.

OI and P (A-a)O_2_ values are essential for evaluating lung ventilation function [27]. A recent study reveals that conventional ultrafiltration does not significantly contribute to the improvement of pulmonary function and reduction in pulmonary complications [28], while a previous study indicates that modified ultrafiltration significantly improved pulmonary compliance and gas exchange compared with the continuous ultrafiltration group [25]. In this study, OI was lower, and P (A-a)O_2_ was higher in both groups after CPB than before CPB, which indicated that cardiopulmonary bypass itself and surgical injury may lead to the decrease of pulmonary ventilation function after surgery. After ultrafiltration, the MUF group’s OI value was higher than the CUF group’s. The MUF group’s P (A-a)O_2_ value was lower than the CUF group’s. The CUF group showed no significant improvement in pulmonary function (Table 2), suggesting that modified ultrafiltration could improve lung function recovery after surgery, consistent with the previous findings.

The extracorporeal circulation of cardiac surgery can induce a systemic inflammatory response [29]. TNF-α is one of the inflammatory factors induced by CPB [30]. Pulmonary function damage after CPB is related to oxygen free radicals in extrapulmonary tissues, neutrophils stimulated by an inflammatory response and TNF-α, which acts as a trigger point in the CPB-induced inflammatory response [31]. IL-6 is a sensitive indicator reflecting the activation of inflammatory response. It is currently believed that the early plasma IL-6 concentration after CPB is closely related to the prognosis of patients [32]. Previous studies have also demonstrated that ultrafiltration can significantly reduce the levels of harmful inflammatory mediators after CPB [31] [33], which also benefits the recovery of patients post-surgery. This study found that IL-6 and TNF-α levels in the MUF group were reduced compared with the CUF group (Figure 3). The results indicated that modified ultrafiltration could further filter out inflammatory factors in the body after CPB shutdown, consistent with the previous findings.

## Conclusion

MUF can improve the efficacy of ultrafiltration, reduce the postoperative systemic inflammatory response, attenuate the lung injury caused by CPB, and improve patients’ lung function in the early postoperative period. It is clinically significant for promoting recovery after cardiac surgery and shows the potential to manage heart valve replacement.

## Dodatak

### Acknowledgements

Not applicable.

### Funding

Not applicable.

### Ethical consideration

The Ethics Committee of the Tangshan Workers Hospital approved this study.

### Author contribution

Geng Ning: Conceptualization, data analysis, drafting the manuscript; Lili Fu: data collection and analysis, revision; Guangwei Zhou: data collection and analysis, revision. All authors read and approved the final manuscript.

### Conflict of interest statement

All the authors declare that they have no conflict of interest in this work.
